# Sir William Jenner

**Published:** 1898-12-17

**Authors:** 


### The Hospital

A Journal of

The Medical Sciences and Hospital Administration.

Vol. XXV.—No. 638.] Edited by Sir Henry Burdett, K.C.B., and Solomon C. Smith, M.D., M.R.C.P. [Dec. 17, 1898.

### Sir William Jenner.

The death of the great physician, who for so many years was the confidential adviser, in all matters within his province, of Her Majesty and of the Royal Fam'ly, is an event which cannot be passed over without a tribute to his memory, or without an attempt to analyse and trace out the sources of his greatness. Her Majesty's own description of him as "a true and devoted friend" will of itself account for much; but the truth and the devotion owed no little of the force which they exercised, and of the confidence which they inspired, to the groundwork of conscientious and untiring labour upon which, as a foundation, they reposed. Above and beyond all other desires and ambitions, Jenner "wanted to know." His career, from its very outset, was governed by his determination to search out the secrets of Nature, and to unravel the processes of disease. Sixty-one years have passed away since he obtained the license of the Society of Apothecaries, and commenced practice in Aldersgate Street, where, if we have not been misinformed, it was necessary for him to eke out his slender professional earnings by keeping a little shop for the sale of sundry small articles commonly supplied by druggists. It does not need a very extensive knowledge of human nature to realize that any ordinary man, finding himself in such a position, would have accommodated his life to his surroundings, and would have remained in the groove which circumstances had moulded for his reception. Jenner, on the contrary, continued to frequent the wards and the post-mortem room of University College Hospital, at which he had been a student, and worked at clinical and pathological problems even more strenuously, after he had obtained a qualification, than he had done in order to obtain it. It is not probable that his early school education had been very deep or very extensive, but his industry made amends for all deficiencies, and, seven years after commencing practice, he graduated as M.D. at the University of London. Four years later he became a Member of the Royal College of Physicians, and the authorities of the hospital, at which his powers and attainments had become well known, gladly availed themselves of the first opportunity to secure him as a colleague. He was appointed Assistant Physician to the hospital, and Lecturer on Pathological Anatomy, his favourite subject, in the school. In 1852 he was elected a Fellow of the college of which, in after years, he was so distinguished a president; and other hospital appointments were from time to time offered to him. He became Physician to the Hospital for Sick Children and to the London Fever Hospital, in which so much of the chief work of his life was done. In all these positions he displayed the same qualities which had first raised him from comparative obscurity. His examinations of patients left nothing uninvestigated, his keenness of observation left nothing unnoticed. Few spectacles could be more interesting than to see Jenner engaged in the analysis of a case which had presented difficulties to others, and which required him to bring to bear upon it all the resources of his unrivalled skill, and of his unequalled experience. Nor, in relation to the sick, was his kindness less manifest than his sagacity. In ordinary life he was not what would be called amiable, and he had a constitutional intolerance of any form of opposition; but, when he stood by the bedside, it was as much his care to cheer the mind of the patient as to relieve his sufferings, or to obviate his danger. In his wards at the hospital no one was ever more considerate or more anxious to avoid the use of any words likely to add to the sufferings which it was at once his business and his pleasure to relieve. In the presence of illness, rank was forgotten, and the prince could receive no more tenderness than was habitually accorded to the peasant. No one ever more thoroughly realised the noble words of Terence, *Humani nihil a me alienum puto*, or more completely carried the principle underlying them into his daily thought and his daily work. It was in one sense his misfortune to have been completely engrossed by medicine, for the time of his retirement found him but slenderly supplied with substitutes for the forms of intellectual activity which had sufficed for him during the more active period of his career. But it may be written upon his tombstone that "he never turned his face from any poor man." Throughout more than half a century of a career which was honourable, not only to himself, but also to the profession which he adorned, he never either neglected his duties for the sake of ease, or abused his opportunities for the sake of gain. May his mantle fall upon successors as able and as worthy as himself.

